# Addressing the mysterious respiratory illness outbreak in China

**DOI:** 10.1016/j.nmni.2023.101206

**Published:** 2023-12-04

**Authors:** Gbolahan Olatunji, Emmanuel Kokori, Abdulrahmon Akanmu Moradeyo, Ibukunoluwa Ogunbowale, Israel Charles Abraham, Bisola Aina, Blessing Chiamaka Ezenwa, Olarewaju Obafemi, Nicholas Aderinto

**Affiliations:** Department of Medicine and Surgery, University of Ilorin, Ilorin, Nigeria; Department of Medicine and Surgery, Ladoke Akintola University of Technology Teaching Hospital, Ogbomoso, Nigeria; Babcock University Teaching Hospital, Ogun, Nigeria; Department of Medicine and Surgery, University of Ilorin, Ilorin, Nigeria; Stoke Mandeville Hospital, Aylesbury, Buckinghamshire, United Kingdom; Department of Medicine and Surgery, Ladoke Akintola University of Technology Teaching Hospital, Ogbomoso, Nigeria

**Keywords:** Outbreak, Respiratory illness, China, Global health

In December 2019, a cluster of pneumonia cases with an unknown aetiology surfaced in Wuhan, Hubei province, China, exhibiting clinical manifestations akin to viral pneumonia [1]. Subsequent investigations by health authorities identified the aetiology as a novel coronavirus, named severe acute respiratory syndrome coronavirus 2 (SARS-CoV-2), leading to the outbreak now known as COVID-19 [1]. Almost four years later, in mid-October of 2023, a surge of influenza-like illness has emerged among children in northern China [2]. This mysterious respiratory illness, characterized by symptoms such as fever, cough, and respiratory distress, demands urgent investigation to determine its aetiology and guide effective containment measures [2].

Chinese authorities have attributed this surge to the lifting of COVID-19 restrictions and the onset of the cold season, pointing to known pathogens like influenza, Mycoplasma pneumoniae, respiratory syncytial virus (RSV), and SARS-CoV-2 as potential causes [2]. The rapid spread and potential severity of this mysterious respiratory illness among children present a significant public health challenge, necessitating immediate identification of the causative agent and implementation of robust containment measures. However, detailed information is currently limited regarding the overall risk of these reported cases [3]. Addressing this outbreak is crucial to safeguard public health, curb further transmission, and mitigate potential long-term consequences on affected children and the community.

The transparency exhibited by the Chinese government and the WHO in disseminating information about the mysterious respiratory illness is crucial. Drawing comparisons to the initial stages of the COVID-19 outbreak, concerns arise about the accuracy of data and the need for further research to determine the connection to earlier respiratory infections. The WHO has formally requested additional epidemiological, clinical, and laboratory data about the outbreaks among children [4]. China's surveillance systems, including those for influenza-like illnesses, contribute to global data collection, but the WHO emphasizes the importance of ongoing surveillance and advises precautionary measures for residents in China [4].

In the face of the current respiratory outbreak in China, urgent collaborative efforts are crucial to contain and swiftly resolve the situation. Drawing from the lessons of the early stages of the COVID-19 pandemic, a comprehensive set of policy recommendations is essential to mitigate the spread of the illness and address potential socioeconomic implications. Enhanced immunization strategies should be prioritized, especially among children aged five and below. Collaborative efforts with international health organizations should ensure the availability and accessibility of vaccines, emphasizing routine vaccinations alongside specific measures targeting the current outbreak. Similarly, advocacy for the widespread use of personal protective equipment (PPE) is paramount. Ensuring an adequate supply of PPE through national and international partnerships is crucial to support healthcare workers, the general population, and those in high-risk environments.

Public health education and communication play a vital role. Launching campaigns to educate the population on the importance of preventive measures, including proper hand hygiene, mask usage, and social distancing, is essential. Utilizing various communication channels, such as social media and community engagement, is crucial for disseminating accurate information. Moreover, global surveillance and collaboration should be strengthened. This involves international collaboration in monitoring and sharing data related to respiratory illnesses, fostering a coordinated response to emerging outbreaks. Facilitating information exchange between countries enables a more rapid and comprehensive understanding of the mysterious respiratory illness in China.

Transparent reporting and data sharing are important. Emphasizing transparent reporting by national health authorities and encouraging prompt and comprehensive sharing of epidemiological and clinical data, as well as laboratory results, ensures a better understanding of the situation globally. As the world continues to recover from the impact of the COVID-19 pandemic, sustaining effective containment measures based on WHO recommendations is imperative to prevent the further spread of the mysterious respiratory illness in China. Urgent collaborative efforts, guided by evidence-based policies, will not only safeguard public health but also contribute to global resilience in the face of emerging health challenges.

## Data statement

No new datasets were generated for this paper.

## Funding

No funding was received for this paper.

## CRediT authorship contribution statement

**Gbolahan Olatunji:** Conceptualization. **Emmanuel Kokori:** Writing – review & editing. **Abdulrahmon Akanmu Moradeyo:** Writing – review & editing. **Ibukunoluwa Ogunbowale:** Writing – review & editing. **Israel Charles Abraham:** Writing – review & editing. **Bisola Aina:** Writing – review & editing. **Blessing Chiamaka Ezenwa:** Writing – review & editing. **Olarewaju Obafemi:** Writing – review & editing. **Nicholas Aderinto:** Writing – review & editing.

## Declaration of competing interest

The authors declare no conflict of interest.
